# Correlation of Country Characteristics and Government Response Measures With COVID-19 Mortality During the First Phase of the Global COVID-19 Pandemic: A Worldwide Ecological Study

**DOI:** 10.7759/cureus.18689

**Published:** 2021-10-11

**Authors:** Ludmil V Mitrev, Annesha Banerjee, Noud Van Helmond

**Affiliations:** 1 Anesthesiology, Cooper University Hospital, Camden, USA; 2 Medicine, Cooper Medical School of Rowan University, Camden, USA

**Keywords:** covid-19, mortality, public health, socioeconomic determinants of healthcare, pandemic

## Abstract

Introduction

It is valuable to know if country demographic, educational, healthcare and other socioeconomic factors were correlated with the COVID-19 mortality rate during the initial phase of the coronavirus disease 2019 (COVID-19) worldwide pandemic (January 1^st^ - August 31^st^, 2020). Similarly, it is worthwhile understanding whether a country’s geographic location or the measures instituted by governments, such as lockdowns and mask-wearing, were associated with an increased or decreased mortality rate.

Materials and methods

To assess these correlations, we conducted an ecologic study of 178 countries using time-matched data from the Social Progress Index (www.socialprogress.org, produced by the Social Progress Imperative), population data from the World Bank (data.worldbank.org), government response indices from Our World In Data (ourworldindata.org/policy-responses-covid), and COVID-19 mortality data from the Johns Hopkins University CSSE COVID-19 Data repository (github.com/CSSEGISandData/COVID-19), accessed on November 22^nd^, 2020. Pearson correlation coefficients were derived between potential predictors and countries’ COVID-19 population-adjusted crude mortality rates. Select variables were entered in a multivariable regression model. Countries with no data in the social progress index database or those with no COVID-19 cases were excluded (20 in total).

Results

The highest positive correlations were found between the proportion of the population older than 75 (Pearson correlation coefficient 0.321), country distance from the equator (0.267), gross domestic product per capita (0.218), health and wellness score (0.388), water and sanitation score (0.384), environmental quality (0.237), and the days between the first reported COVID-19 case and the initial government response (0.238). A previously unreported and unexpected negative correlation was found between gender parity in secondary education attainment and COVID-19 mortality (-0.290). Peak mask-wearing ranging from ‘recommended’ to ‘required outside the home at all times was extremely weakly correlated with lower COVID-19 mortality (-0.046).

Conclusions

Crude COVID-19 mortality rates during the first phase of the pandemic in 2020, during which no vaccine or specific treatment was available, were higher in wealthier countries that were further away from the equator and had a higher health and wellness score according to the Social Progress Imperative. They were also higher the longer governments delayed their initial response. Gender parity in secondary education and stringency of mask-wearing guidelines were correlated with lower mortality, though the latter correlation was extremely weak. Our findings are consistent with previously published correlations. The correlation between crude COVID-19 mortality rates and gender parity in secondary education has not been previously reported.

## Introduction

The novel coronavirus disease 2019 (COVID-19) and the resulting worldwide pandemic require no introduction. Virtually every country has been affected. Some countries experienced several waves of the infection before the institution of vaccination in late 2020/early 2021, and some continue to struggle with novel variants of the severe acute respiratory distress syndrome-coronavirus-2 (SARS-CoV-2), causing increased infection rates and mortality. SARS-CoV-2 is transmitted mainly via respiratory droplets [1], and until the advent of specific vaccines, social distancing, mouth and nose coverings and hand-washing were the only modes of protection against the spread of the virus. The arch-model of emerging and re-emerging viral respiratory pandemics are the influenza pandemics of the past 150 years [2]. The history of such pandemics illustrates the importance of understanding not only viral spread, the host immune response, and science’s ability to construct vaccines against the virus, but also society’s ability to restrict the spread of the pathogen and reduce morbidity and mortality during the early stages of the pandemic, during which little is yet known about the pathogen and no specific treatment is available.

To investigate country socio-economic and educational/knowledge factors and their correlation to crude COVID-19 mortality rates, we conducted an epidemiologic study involving all of the world’s countries during the initial phases of the pandemic from January 2020 to August 31st, 2020. Several studies using broadly similar methods were conducted in 2020 but focused on a limited number of countries, did not evaluate socioeconomic or educational factors, or were limited to the very early stages of the pandemic in 2020 when the mortality curve was rising [3-7]. We felt it was necessary to include all countries in our analysis using a broader range of independent variables covering the entire first wave of the global pandemic in 2020.

Our primary hypothesis was that higher wealth of countries, higher health and wellness scores and higher degrees of educational attainment were associated with lower COVID-19 mortality. Our co-primary hypothesis was that the speed and stringency of the initial governmental response, measured in days from the index case in each country and the degree of mandatory face coverings, was associated with lower COVID-19 mortality.

## Materials and methods

To assess these correlations, we conducted an ecological study of all countries using time-matched data from the Social Progress Index (SPI, www.socialprogress.org, produced by the Social Progress Imperative) [8], population data from the World Bank (data.worldbank.org), government response indices from Our World In Data (ourworldindata.org/policy-responses-covid), and COVID-19 mortality data from the Johns Hopkins University CSSE COVID-19 Data repository (github.com/CSSEGISandData/COVID-19) accessed on November 22nd, 2020. Countries with no data in the social progress index database or those that reported no COVID cases in the study period were excluded from analysis (n=20 in total). Therefore a total of 178 countries were analyzed. Data was retrieved for the period January 1st - August 31st, 2020.

**Figure 1:**
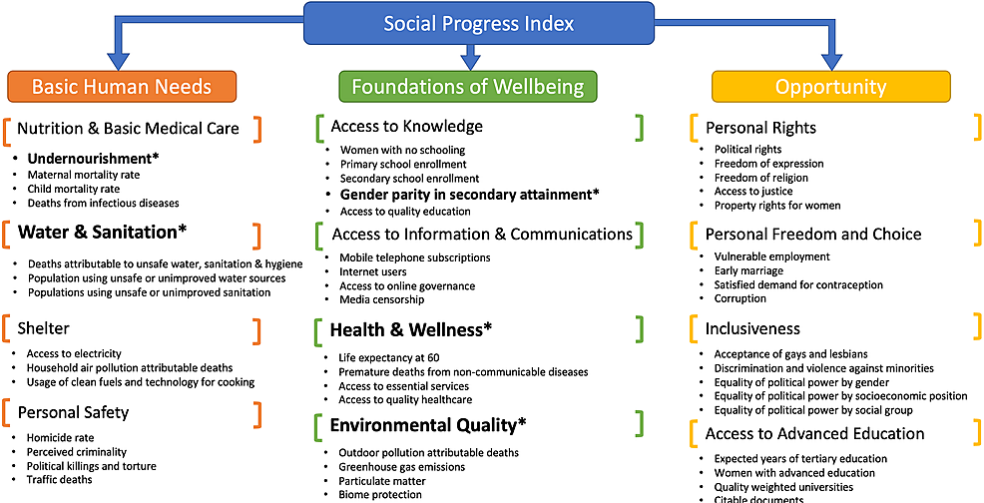
Composition of the Social Progress Index (SPI). All dimensions, components and indicators are listed. Component and indicator scores used in the analysis are indicated with an asterisk.

The Social Progress Index (SPI) is an established measure of countries’ wellbeing that has been designed as a holistic, transparent and outcome-based benchmark independent of economic indicators. The SPI allows between-country comparisons in three main dimensions: (1) basic human needs; (2) foundations of wellbeing; and (3) opportunity. Each of these encompasses four components, which in turn are based on three to five indicators. Fifty indicators in total are used to calculate the overall SPI, its dimension and component scores [9]. Figure 1 represents the SPI’s indicator level framework, adapted from www.socialprogress.org (Figure 1) [8,9]. 

The ‘Health and wellness’ component score is a composite calculated from data on life expectancy at 60 years, premature deaths from non-communicable diseases (deaths/100,000), access to essential services (0=none; 100=full coverage) and access to quality healthcare (0=unequal; 4=equal). The ‘Environmental quality’ component of SPI comprises outdoor air pollution attributable deaths (deaths/100,000), greenhouse gas emissions (total CO2 equivalents), particulate matter and biome protection. The ‘Water and sanitation’ component score is derived from unsafe water, sanitation and hygiene attributable deaths (per 100,000 population), populations using unsafe or unimproved water sources (%) and populations using unsafe or unimproved sanitation (%). Undernourishment (% of population) and gender parity in secondary education attainment (distance from parity) are individual indicators within SPI dimensions 1 and 2, respectively.

The geographical latitude of countries was based on rounded latitude figures for the purpose of finding the approximate geographic center of an entity, as published in the Gazetteer of Conventional Names [10]. The proportion of a country’s population > age 75 was obtained from United Nations data [11]. Labor force participation rate (% > 15 years of age) was obtained from World Bank data [12]. Population density and GDP were sourced from United Nations data [13]. Policy responses to the coronavirus pandemic were obtained from Our World In Data, a project of Global Change Data Lab in collaboration with the University of Oxford [14]. Peak mask-wearing was scored ordinally from ‘recommended’ (0) to ‘required outside the home at all times (4).

Statistical analysis

Individual countries were the unit of analysis and country characteristics were presented descriptively as mean with standard deviation. The primary outcome was crude mortality, expressed as confirmed deaths normalized to deaths per million of each country’s population. We aimed to build a multiple regression model to predict the primary outcome with the retrieved country general characteristics, socioeconomic characteristics, health and healthcare-related characteristics, environmental and hygiene characteristics, and governmental COVID-19 responses. We assessed multicollinearity among predictor variables and the SPI scores used were found not to be significally intercorrelated. An initial univariate screening was used to identify factors with a linear relationship with COVID-19 mortality. A Pearson ρ of (-) 0.2 was used as a threshold to remove weak predictors. Selected variables were then entered into a multivariable regression model. We used best subset selection to determine the final set of independent variables for retention in the COVID-19 outcome model. We used the adjusted R^2^ to select a model. Reported results of the regression analysis were regression coefficients with confidence interval, standardized coefficients, R^2^, adjusted R^2^, and predicted R^2^. Normality of residuals and homoscedasticity were assessed by plotting regression residuals. All the statistical analyses were performed using JMP Pro (SAS, Cary, NC, USA). 

## Results

Table 1 lists the Pearson correlation coefficients of the tested variables. 

**Table 1:** Average country characteristics and associations with death rate (COVID-19 deaths/population/year). *Correlation coefficient represents the Pearson correlation coefficient between potential predictors and countries’ COVID-19 crude mortality rate per year. COVID-19: coronavirus disease 2019; GDP: gross domestic product per capita in U.S. dollars. Coefficients exceeding 0.2 are listed in bold typeface.

	Mean ± SD	Correlation coefficient*
General characteristics		
Proportion of population over age of 75 years in percent	3.7 ± 3.2	0.321
Latitude of capital city in degrees from the equator	27 ± 17	0.267
Population density in persons per square kilometer	366 ± 2076	-0.015
Socioeconomic characteristics		
GDP	16,759 ± 26,596	0.218
Labor force participation in percent	63 ± 10	-0.094
Gender parity in secondary education attainment in distance from parity (0-1)	0.15 ± 0.17	-0.290
Undernourishment in percent	9 ± 10	-0.262
Health and healthcare-related characteristics		
Health and wellness score (0-100 scale)	63 ± 16	0.388
Environmental and hygiene characteristics		
Water and sanitation score (0-100 scale)	76 ± 25	0.384
Environmental quality (0-100 scale)	75 ± 13	0.237
Government COVID-19 response		
Government stringency index peak response (0-100 scale)	83 ± 14	0.072
Days between first case and initial stringency response	-19 ± 24	0.238
Days between first case and peak stringency response	39 ± 32	0.138
Peak face-covering policies response (0-4 scale)	2.8 ± 1.0	-0.046
Days between first case and initial face-covering response	62 ± 50	0.021
Days between first case and peak face-covering response	84 ± 55	0.094

The highest positive correlations were found between the proportion of the population older than 75 (Pearson correlation coefficient 0.321), country distance from the equator (0.267), gross domestic product per capita (0.218), health and wellness score (0.388), water and sanitation score (0.384), environmental quality (0.237), and the days between the first reported COVID-19 case and the initial government response (0.238). A previously unreported negative correlation was found between gender parity in secondary education attainment and COVID-19 mortality (-0.290). Peak mask-wearing was extremely weakly correlated with lower COVID-19 mortality (-0.046).

**Figure 2:**
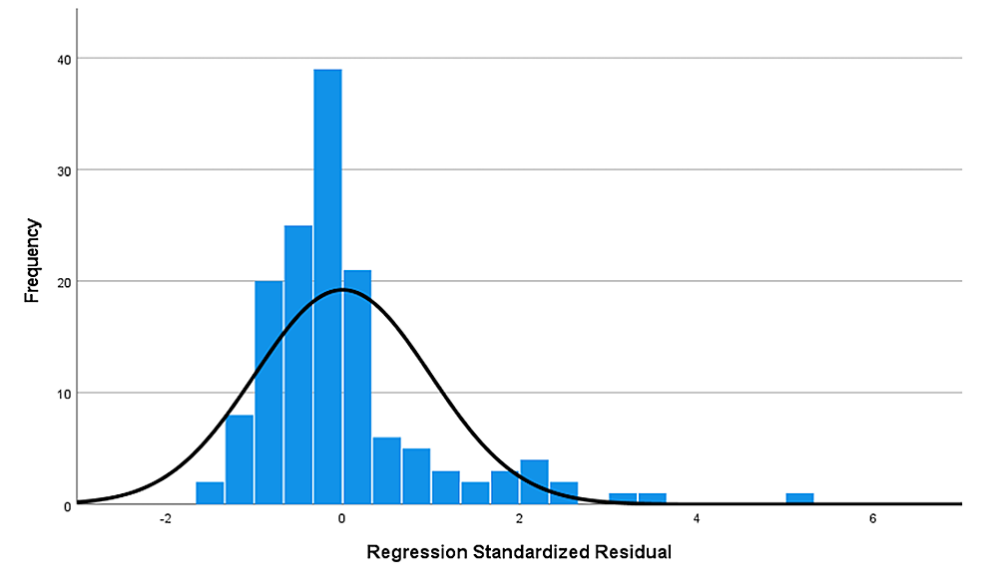
Histogram of standardized residuals. The mean (-7.25 x 10-16) and standard deviation (0.989) approximated 0 and 1, respectively, indicating that the standardized residuals were approximately normally distributed.

Table 2 represents the multiple regression model. If the correlation coefficient was higher than 0.2, we considered it potentially significant and those variables were entered in the multiple regression model. A histogram of the standardized residuals in the model is shown in Figure 2.

**Table 2:** Regression results for crude COVID-19 death rate. B = unstandardized regression coefficient; CI = confidence interval; SE B = standard error of the coefficient; β = standardized coefficient; R^2^ = coefficient of determination; 𝛥R^2^ = adjusted R^2^. GDP, Gross domestic product per capita in U.S. dollars.

COVID-19 death rate	B	95% CI for B	SE B	β	R^2^	𝛥R^2^
Model					0.19	0.18
Constant	-251	-510 – 8.0	131			
GDP	2.9 x 10^-4^	-3.2 x 10^-3 ^– 3.7 x 10^-3^	1.7 x 10^-3^	0.018		
Days between first case and initial stringency response	1.7	-3.9 x 10^-1 ^– 4.0	1.1	0.127		
Health and wellness score (0-100 scale)	7.7	3.0 – 12.0	2.2	0.378		

Only GDP, the number of days between the first confirmed COVID-19 case and the government’s initial response, and the health and wellness score were retained in the final model. The overall model was statistically significant (F (4, 95) = 9.0, P < 0.0001). The overall adjusted coefficient of determination of the final model (R^2^) for crude death rate was low (0.18). The predicted R^2^ was 0.12.

## Discussion

None of the correlations found were strong, which was expected given the nature of the study. Some of our findings were directionally similar to other reported correlations in the literature, whereas others were unexpectedly opposite in direction. For instance, Sorci et al. reported that the Covid fatality rate was highest in countries with high GDP [6]. Pana et al. also found that GDP per capita had a statistically significant relationship with log mean Covid mortality rate in their univariable analysis [5]. De Larochelambert et al.’s correlation matrix showed that the COVID-19 mortality rate was positively correlated to GDP, obesity, burden of mortality due to chronic diseases such as cardiovascular disease or cancer, life expectancy, economic support index, and deviation from latitude zero [7]. Our analysis corroborates the findings that wealthier countries with older populations that are further away from the equator had higher COVID-19 population-adjusted crude mortality rates. This may have been due to the comorbidities of aging and a greater likelihood to congregate indoors in cooler climates. Another possible explanation for the association with GDP could be that less wealthy countries had fewer resources available for testing, thereby underestimating their COVID-19 mortality.

Global burden of disease estimates from the World Health Organization show that persons >60 years of age account for 49.2% of the global disease burden in high-income regions, but 19.9% in low-income regions, and that the leading contributors to disease in older populations are chronic non-communicable diseases such as cardiovascular ailments [15]. This uneven distribution of chronic disease burden could be one explanation for our finding that richer countries, as expressed by GDP, had a higher crude mortality rate. Another possible explanation could be the greater mobility of populations in such countries, which in turn may have been an important factor for viral spread.

Unsurprisingly, the longer it took governments to mount a stringent response to the pandemic, the greater the COVID-19 crude mortality rate. The stringency of face-covering policies, however, appeared to have been negligibly correlated with lower mortality. 

The following associations were directionally opposite to what one might have intuited. We expected countries with higher health and wellness scores to have suffered a lower mortality. The SPI health and wellness score encompasses data on life expectancy at 60 years, mortality due to cardiovascular diseases, cancers, diabetes, and chronic respiratory diseases in those aged 30-70 years, universal health coverage, and equal access to healthcare. Countries with higher composite health and wellness scores may have had lower morbidity and mortality from the aforementioned conditions, but this would have improved the life expectancy of their citizens and likely reflects better management of chronic disease rather than its absence. This supports the previously published findings that mortality from COVID-19 is highest in the elderly and those suffering from chronic ailments [16]. The incidence of diabetes, hypertension, cardiovascular disease, chronic obstructive pulmonary disease and malignancies are all higher in elderly populations [15].

Undernourishment appears to have been negatively correlated with COVID-19 mortality, but this association could have been mediated via lower rates of obesity, hypertension and/or diabetes. Higher gender parity in secondary education attainment likewise appeared to have been protective. Having more educated women in countries may have translated to a higher adherence to healthcare safety guidelines for themselves, their children and the elderly. Women traditionally tend to provide more care to those demographic groups on a worldwide basis.

This study suffers from the well-known shortcomings of ecologic studies [17]. The concept of “ecologic fallacy” or bias refers to the detection of associations of risk factors and outcomes on a country or community level without knowing whether each citizen or member of the group was exposed to the risk factor and had a measured outcome. Ecologic bias can only be avoided by assessing individual-level information, which was not possible in our study. Nevertheless, the country socio-economic and governmental response data varied largely between countries, and the rapid spread of the SARS-CoV-2 virus led to ramped-up testing and assiduous mortality reporting of COVID-19 deaths in most countries, thereby providing a unique opportunity for our study to be conducted.

## Conclusions

It is of critical importance for governments to know which aspects of their responses and socioeconomic/healthcare system strengths would be associated with better outcomes in a respiratory viral pandemic, at a time when no vaccine or specific treatment is available. This may inform future pandemic preparedness. Our ecologic study found weak positive correlations between crude COVID-19 mortality in countries and the following: the proportion of the population older than 75, country distance from the equator, gross domestic product per capita, health and wellness score, water and sanitation score, environmental quality, and the days between the first reported COVID-19 case and the initial government response to the pandemic. A previously unreported negative correlation was found between gender parity in secondary education attainment and COVID-19 mortality. Peak mask-wearing ranging from ‘recommended’ (0) to ‘required outside the home at all times’ (4) was extremely weakly correlated with lower COVID-19 mortality. Our multiple regression model retained only GDP, the number of days between the first confirmed COVID-19 case and the government’s initial response, and the health and wellness score. While the multiple regression model was statistically significant, it had a low coefficient of determination for the crude death rate. Our finding of a correlation between gender parity in secondary education and crude COVID-19 mortality is novel and deserves further investigation.
